# Submaximal Verification Test to Exhaustion Confirms Maximal Oxygen Uptake: Roles of Anaerobic Performance and Respiratory Muscle Strength

**DOI:** 10.3390/jcm13195758

**Published:** 2024-09-27

**Authors:** Kamil Michalik, Natalia Danek

**Affiliations:** 1 Department of Human Motor Skills, Faculty of Physical Education and Sport, Wroclaw University of Health and Sport Sciences, 51-612 Wroclaw, Poland; 2 Department of Physiology and Biochemistry, Faculty of Physical Education and Sport, Wroclaw University of Health and Sport Sciences, 51-612 Wroclaw, Poland; natalia.danek@awf.wroc.pl

**Keywords:** incremental test, ramp protocol, verification phase, anaerobic capacity, respiratory muscles fatigue

## Abstract

**Background**: The incremental exercise test is commonly used to measure maximal oxygen uptake (VO_2_max), but an additional verification test is often recommended as the “gold standard” to confirm the true VO_2_max. Therefore, the aim of this study was to compare the peak oxygen uptake (VO_2_peak) obtained in the ramp incremental exercise test and that in the verification test performed on different days at submaximal intensity. Additionally, we examined the roles of anaerobic performance and respiratory muscle strength. **Methods**: Sixteen physically active men participated in the study, with an average age of 22.7 ± 2.4 (years), height of 178.0 ± 7.4 (cm), and weight of 77.4 ± 7.3 (kg). They performed the three following tests on a cycle ergometer: the Wingate Anaerobic Test (WAnT), the ramp incremental exercise test (IET_RAMP_), and the verification test performed at an intensity of 85% (VER_85_) maximal power, which was obtained during the IET_RAMP_. **Results**: No significant difference was observed in the peak oxygen uptake between the IET_RAMP_ and VER_85_ (*p* = 0.51). The coefficient of variation was 3.1% and the Bland–Altman analysis showed a high agreement. We found significant correlations between the total work performed in the IET_RAMP_, the anaerobic peak power (r = 0.52, *p* ≤ 0.05), and the total work obtained in the WAnT (r = 0.67, *p* ≤ 0.01). There were no significant differences in post-exercise changes in the strength of the inspiratory and expiratory muscles after the IET_RAMP_ and the VER_85_. **Conclusions**: The submaximal intensity verification test performed on different days provided reliable values that confirmed the real VO_2_max, which was not limited by respiratory muscle fatigue. This verification test may be suggested for participants with a lower anaerobic mechanical performance.

## 1. Introduction

Maximal oxygen uptake (VO_2_max) has been established as an important indicator of cardiopulmonary fitness and one of the most important determinants of endurance performance. It represents the upper limit for the transport and ability of the muscles to utilize oxygen [1]. VO_2_max measurements refer to aerobic capacity evaluation in the general population, talent identification in endurance sport disciplines, and the examination of the effectiveness of different training programs after long-term interventions (e.g., high-intensity interval training). The incremental exercise test (IET) for evaluating maximal oxygen uptake has many practical applications for healthy populations and athletes [2]. IETs with graded increases in intensity are commonly used in physiological laboratories, where individual stages can last from 1 to 5 min [3], or for ramp protocols with a constant slope of load increase [4,5,6,7]. These ramp protocols provide a constant linear increase in workload in every second [5]. Previous studies by Michalik et al. [5] showed that the peak power and VO_2_max in young road cyclists were higher in the ramp test (0.28 W·s^−^^1^) compared to the step test (50 W·min^−^^1^). Moreover, there was no significant difference in power at anaerobic threshold (AnT) between both tested protocols. In turn, Danek et al. [6] implemented a 15-min warm-up at a 60% VO_2_max intensity before the ramp protocol. They reported a greater VO_2_max, maximal power (Wmax), maximal lung ventilation (VEmax), and maximal heart rate (HRmax) [6]. Despite these findings, further studies are needed to verify linear ramp protocols to measure real VO_2_max.

Physiologists have developed various methodological criteria known as primary and secondary criteria to confirm measurements of real VO_2_max [2,4,8,9]. For instance, the primary criteria include reaching a plateau in oxygen uptake (no further increase in VO_2_ despite the increase in load). The secondary criteria include the following blood lactate concentration ([La^−^]), HRmax, respiratory exchange ratio (RER), and rate of perceived exertion (RPE). These criteria have been fully discussed in several studies [2,4,8,10]. Contrary, it was found that, during reaching VO_2_max, it is not possible to expose the VO_2_ plateau phase [9,11], and the occurrence of this phenomenon presents varied results in the available literature (17–100%) [5,12,13]. On the other hand, using the secondary criteria is problematic [10], because they can be achieved for a submaximal exercise intensity [4]. Additionally, these criteria are characterized by a high individual variability, which reflects the limitations in their application [12,14]. It was also proven that people with a lack of experience in performing these maximal exercise tests, those with low cardiovascular fitness, and those with insufficient motivation can finish incremental exercise tests before reaching VO_2_max due to different symptoms of fatigue [10]. These results can lead to an underestimation of individual physical capacity and lower values of VO_2_max.

In recent years, the concept of a verification test to confirm the actual VO_2_max has emerged. Many studies are summarized in the reviews published by Schaun [9] and Costa et al. [15]. This area of research is attracting attention due to the need to standardize verification protocols and the criteria for achieving real VO_2_max [9]. On the other hand, some authors have questioned whether this approach is appropriate, e.g., due to the proven high compliance of VO_2_max from the incremental exercise and verification tests [15,16,17]. Usually, verification tests last a few minutes [9,15,18] and are performed from 1 to 90 min with a passive or active break after finishing the IET [15,16,19] or on a different day [15,18,20,21]. The intensity of exercise during the VER relative to that achieved in the IET is one of the issues that needs to be considered [20], because there is no clear-cut understanding of the load intensity that should be used to provoke the maximal physiological responses [22]. The most frequently used verification phase is effort with maximal or supramaximal intensity, i.e., 100 ≤ 115% of the peak power obtained in the IET [15,19,20]. It has also been verified that protocols with submaximal intensity from 80 to <100% of peak power provide reliable agreement for VO_2_max [15,16,19,20]. Poole and Jones [10] suggested that the verification phase should be conducted with an intensity exceeding the peak power obtained in the incremental test. In particular, a verification phase with less than 100% peak power should be preferred, as this can be sustained adequately long to allow for the attainment of VO_2_max [22]. These observations were confirmed by the research of Murias et al. [16], who compared VER at intensities of 85% and 105% of peak power carried out 5 min after an IET in young and older participants. The study indicated that the verification phase provided no additional benefit to the VO_2_max measurement from the ramp test. However, there have been no studies assessing whether the use of a lower intensity, e.g., 85% of the maximal workload on another day, would lead to a similar VO_2_max result from the ramp test with a linear increased workload.

The critical requirement for a reliable measurement of VO_2_max is exercise duration, which justifies the implementation of loads above and below the peak power applied during the verification phase [23]. When the exercise time is long enough, due to the presence of a VO_2_ slow component, an exercise intensity exceeding AnT and/or the critical power (CP) should allow for reaching VO_2_max [22], as well as a plateau [15]. This justifies the use of the so-called open loop, i.e., an effort to refuse/volitional exhaustion, which is similar in nature to the incremental exercise test [24]. This also indicates the importance of anaerobic performance, where a high level enables tolerance to fatigue in conditions of metabolic acidosis and reaching a VO_2_ plateau [25,26]. The increasing level of the anaerobic metabolism during an IET [27] can be assessed using indirect markers of glycolysis (the concentrations of hydrogen and lactate ions) [28]. Astorino and White [29] conducted a study that determined the significance of the anaerobic performance measured by the Wingate Anaerobic Test (WAnT) in individuals who confirmed or did not confirm VO_2_max in a supramaximal-intensity verification test conducted 10 min after an IET. Interestingly, participants who did not verify the VO_2_max measured in the IET achieved a 10% higher peak and mean power during the WAnT, which can explain the higher homeostasis disturbances and level of fatigue after an IET. In order to explain these results, conducting a verification test on a different day and measuring the post-exercise lactate concentration and acid–base balance were proposed [29]. This is important, because it is known that increasing the anaerobic metabolism leads to increasing the acute response in lung ventilation and increasing the work of the respiratory muscles. This causes, in a metaboreflex response, a reduction in blood flow through the working limbs and fatigue of the locomotor muscles [30]. It can also be a factor causing fatigue of the respiratory muscles, limiting their performance (loss of strength) during maximum effort, as reported after an incremental treadmill test and a cycle ergometer test [31]. Therefore, further studies are needed to determine to what extent these factors determine the achievement of genuine VO_2_max.

To the best of our knowledge, no previous studies have assessed whether a verification phase with a constant, open-loop task performed on a different day confirms the reliability of the VO_2_max measured in the ramp test. Therefore, to fill this research gap, the main aim of this study was to determine whether the verification test performed on another day at an intensity of 85% of the maximal power obtained in a ramp IET confirms VO_2_max. The second goal was to determine the role of the anaerobic performance measured in the WAnT and compare it between individuals, depending on which test they obtained a higher VO_2_peak in. The third goal was to determine whether respiratory muscle strength decreases after an IET and VER. We formulated the following hypotheses: (1) the VO_2_max will not differ between the tests, (2) the anaerobic performance level will affect the possibility of continuing effort (the total work performed) until volitional exhaustion in these tests, and (3) a post-exercise decrease in respiratory muscle strength in both tests will occur.

## 2. Materials and Methods

### 2.1. Participants

This study involved sixteen healthy, physically active men. All participants were volunteers and declared a minimum five hours of physical exercise (sports classes at the university, gym, volleyball, football, and running) per week. None of the participants trained sports at a professional level or were classified as being at risk of cardiovascular, respiratory, or metabolic diseases. There were no smokers, drug use, or nutritional support use among the participants. All recruited participants were acquainted with the study procedure, gave their written informed consent to participate, and completed the study. The study was approved by the local Research Ethics Committee (1/2019) and was carried out following the Declaration of Helsinki in a laboratory with the certificate PN-EN ISO 9001:2015, Kobierzyce, Poland, 2022. Detailed characteristics of the study participants are presented in Table 1.

The sample size was established a priori using the G*Power 3.1 software (v.3.1.9.2, Heinrich-Heine-Universität Düsseldorf, Düsseldorf, Germany) [32], the expected effect size was set at (Cohen’s f) 0.8, the α level was set at 0.05, and the power (1-β) was set at 0.9 [33]. Sixteen participants in the group were necessarily recruited.

### 2.2. Study Design

**Figure 1:**
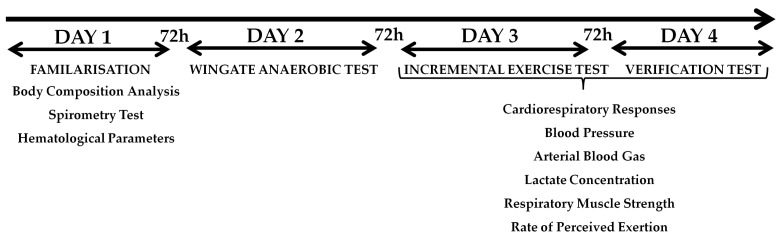
Experimental protocol.

The experimental study included four visits to the laboratory, with a minimum of 72 h between visits. During the first visit, anthropometric measurements, spirometry, hematological parameters, and familiarization with the research procedures were performed. During the second visit, the WAnT was performed for the lower limbs. On the third day, the ramp incremental increase test (IET_RAMP_) was conducted, and on the fourth day, the verification test (VER_85_). Gas analysis, arterial blood gas, lactate concentration, respiratory muscle strength, and rating of perceived exertion were examined during the IET_RAMP_ and the VER_85_. All sessions were administered by the same group of researchers. The participants were asked not to undertake any strenuous effort or to give up training altogether within 24 h before each test. Likewise, they were supposed to avoid caffeine and alcohol consumption during this time. Both tests were performed at the same time of day (08:00 AM–11:00 AM) under laboratory conditions to avoid daily fluctuations in performance. The subjects consumed the same diet during the two days preceding each test to avoid any influence on metabolism. Figure 1 shows the experimental protocol.

### 2.3. Body Composition Analysis

Measurements of height and body weight were made on a WPT 200 medical scale (RADWAG, Radom, Poland), and body composition was measured using the FUTREX analyzer 6100/XL (Futrex Tech, Inc., Gaithersburg, MD, USA). The software then calculated the percentage of body fat (%FAT) based on the near-infrared spectrophotometry (NIRS) method. Before the examination, the participants were instructed to empty their bladder. The probe was placed at the middle of the biceps brachia muscle or the dominant upper limb. All of anthropometry and body composition measurements were performed before the exercise test. The NIRS method has been approved to determine the %FAT in humans [34].

### 2.4. Hematological Parameters

Capillary blood was collected from the fingertip at rest for the determination of the following blood morphotic parameters: hemoglobin concentration (Hb) and hematocrit (Ht) using the ABX Micros OT.16 (Horiba Medical, Kyoto, Japan).

### 2.5. Pulmonary Function Procedure

Spirometry tests were performed using a Quark b^2^ analyzer (Cosmed, Milan, Italy). These tests were conducted in a manner consistent with previous research published by Szczepan et al. [35]. Each test was preceded by air flow calibration. It involved inspiration with a maximum volume preceded by 2–3 quiet breaths and ended with an intense exhalation with a maximum airflow, resulting in a minimum volume of residual air. Each participant performed three trials, and the one with higher FEV_1_ value was selected for further analysis. In the course of the respiratory test, the following parameters were recorded: forced vital capacity (FVC) and forced expiratory volume in 1 s (FEV_1_). The software calculated the Tiffeneau index (FEV_1_·FVC^–1^).

### 2.6. Wingate Anaerobic Test (WAnT)

In order to assess anaerobic performance, the WAnT for the lower limbs was performed [36]. The test was performed on an Ergomedic E894 cycle ergometer (Monark Exercise AB, Varberg, Sweden). The cycle ergometer was calibrated before starting the tests. Then, a warm-up was performed, as recommended by the Bar-Or [36]. This warm-up lasted 5 min and consisted of pedaling with a 2% body weight resistance, separated by three short 5 s maximal sprints. After warming up, the subjects remained in a sitting position on the cycle ergometer for five minutes. The flywheel load was 7.5% of the subject’s body weight. The effort lasted thirty seconds and the task of the participant was to perform the work with the maximum (possible) cadence to achieve the peak power output as quickly as possible, maintaining this until end of the test. The subjects were motivated by verbal encouragement in order to exercise as much effort as possible. The cycle ergometer was controlled by a computer and the MCE v.2.3 software (MCE, Wroclaw, Poland). The peak power output (PPO), relative peak power (rPPO), total work (TW_WAnT_), and fatigue index (FI) were calculated.

### 2.7. Incremental Exercise Test (IET_RAMP_)

The incremental exercise test was carried out on an Excalibur Sport cycle ergometer (Lode BV, Groningen, The Netherlands) according to the protocol described by Michalik et al. [5]. It started with a 0 W load, which increased every second by another ~0.278 W (corresponding to 50 W·3 min^−1^). The minimum cadence was set at 60 rpm. The effort continued until volitional exhaustion. The maximal workload (Wmax) was calculated as the multiplication of the test duration (s) and the load-increasing coefficient. The total work performed (TW_IET_) was calculated based on the obtained maximal power and the test duration [5].

### 2.8. Verification Test (VER_85_)

The verification test was performed on the same Excalibur Sport cycle ergometer (Lode BV, Groningen, The Netherlands). Before the test, a 10-min warm-up was performed with an intensity corresponding to 40% Wpeak with the IET_RAMP_. The task was to maintain the cadence in the range of 60–90 rpm^−1^. The subject then waited for 5 min in a sitting position to begin the main part of the test. The test was carried out at a constant load of 85% of the maximal power obtained in the ramp IET (85% Wmax) and continued until the cadence dropped to below 60 rpm^−1^. The work performed during the test (TW_VER85_) also was calculated.

### 2.9. Cardiorespiratory Response Analysis

The subjects breathed through a mask and the expired gas was analyzed by a Quark b^2^ device (Cosmed, Milan, Italy). The device was calibrated with atmospheric air and a gas mixture with the following composition: CO_2_—5%, O_2_—16%, and N_2_—79%, before starting the measurements. The breathing parameters were registered in each breath (breath by breath). Lung ventilation (VE) and its components, respiratory rate (Rf) and tidal volume (VT), oxygen uptake (VO_2_), and carbon dioxide output (VCO_2_), were measured. The results were averaged every 30 s and converted into minute values to exclude false breaths caused by coughing, gasping, or swallowing. Reducing “noise” and artefacts can improve data interpretation. VO_2_max was recorded as the highest 30 sec mean value during the VO_2_ plateau at the end of exercise or if at least two of the following criteria were met: (1) volitional exhaustion, (2) heart rate (HR) ≥ 95% of age-predicted maximal heart rate (HRmax) (208–0.7·age), and (3) respiratory exchange ratio ≥1.10. The oxygen uptake plateau was determined using predetermined methods and specified as the period during which the VO_2_ did not fluctuate ≤1.5 mL·kg^−1^·min^−1^ after reaching VO_2_max [5]. Heart rate measurements were carried out using the RS400 sport-tester (Polar Electro OY, Kempele, Finland) and were recorded by the software of the Quark b^2^ analyzer.

### 2.10. Blood Pressure and Stroke Volume

Arterial blood pressure was measured at rest before starting and immediately after completing the tests using an aneroid sphygmomanometer (Riester, Jungingen, Germany). Stroke volume (SV) was estimated by two independent methods. The first method (1) is based on post-exercise blood pressure measurements and the Jackson formula [37]:

SV_1_ = 101 + (0.5·PP) − (0.59·DP) − (0.61·age) (1)

where SV—stroke volume (mL), PP—pulse pressure, the difference between systolic and diastolic pressure (mm Hg), and DP—diastolic pressure (mm Hg), age (in years). The second method (2) is based on Fick’s law and is dependent on the VO_2_ and HR when VO_2_max is achieved during the incremental exercise test [38]:

SV_2_ = ((VO_2_peak·16.22^−1^)·HR^−1^)·100 (2)

where SV—stroke volume (mL), VO_2_peak—peak oxygen uptake in the incremental exercise test (mL min^−1^), and HR—heart rate at VO_2_peak in the graded exercise test (beats min^−1^). Both methods have previously been used to verify post-training changes in VO_2_max [39].

### 2.11. Arterial Blood Gas and Lactate Concentration

Capillary blood was collected from the fingertip (before the test, at rest, and in the third minute after finishing, and in the case of VER_85_, also immediately after warm-up) to determine the acid–base balance using the RapidLab 348 analyzer (Bayer, Leverkusen, Germany). The results include blood pH data. The blood lactate concentration ([La^−^]) was also measured on the photometer LP 400 (Dr Lange, Berlin, Germany).

### 2.12. Respiratory Muscle Strength Analysis

Inspiratory muscle strength (maximal inspiratory pressure [PImax]) and expiratory muscle strength (maximal expiratory pressure [PEmax]) were measured in a test using a Micro RPM respiratory pressure meter (CareFusion, San Diego, CA, USA). Dimitriadis et al. [40] reported that Micro RPM reliably measured PImax and PEmax. To assess PImax, the tested person, in a standing position, performed maximum inspiration from the level of maximum expiration. Then, to evaluate PEmax, the individual exhaled starting from the maximum inspiration level. In both cases, a special stopper was fitted. The PImax and PEmax tests were conducted at rest (PImax_PRE_ and PEmax_PRE_) and five minutes after finishing each test (PImax_POST_ and PEmax_POST_). The participants performed two trials for maximum inspiration and maximum expiration each, and the higher values were selected for further analysis.

### 2.13. Rate of Perceived Exertion

The Borg scale [41], which consists of 15 levels (6–20), was used to assess the subjective feeling of effort described as the rate of perceived exertion (RPE). The subjects evaluated their perceived effort immediately after the warm-up and exercise tests. The indicated scale values were submitted for further statistical analysis.

### 2.14. Statistical Analysis

The IBM SPSS Statistics version 26 software package (IBM, Inc., Chicago, IL, USA) was used for statistical analysis. The results are presented as arithmetic mean ± standard deviation ($\bar{x}\pm SD$) and 95% confidence intervals (95% CI). The Shapiro–Wilk test was used to assess the normality of the distribution of the examined features and the homogeneity of variance was assessed using Levene’s test. The paired Student’s *t*-test was used to evaluate the differences between the protocols. The Mann–Whitney U test was used to evaluate the differences in the parameters measured with the WAnT between the groups that reached VO_2_peak in the IET_RAMP_ or VER_85_, respectively. The Pearson’s correlation coefficient between the variables obtained in the WAnT and the work performed during the IET was calculated. To determine the practical implications, the effect size (ES) was calculated as Cohen’s d according to the following criteria: 0.1—trivial, 0.2—small, 0.5—medium, and 0.8—large [33]. A Bland–Altman plot analysis was performed if no significant difference in VO_2_peak was observed. Limits of agreement (LoA) were used to compare individual differences between variables. Mean differences ± 1.96 SD were provided for LoA lines. The coefficient of variation (CV) for VO_2_peak was calculated. A two-way analysis of variance (ANOVA) with repeated measurements was performed to determine the strength of the expiratory muscles. The post hoc Bonferroni test was performed when a significant F ratio was obtained. The effect size was calculated as partial eta-square (η^2^) (small ≥ 0.01 to ≤0.06, moderate ≥ 0.07 to ≤0.13, and large ≥ 0.14) [42]. A *p* ≤ 0.05 level was considered to be statistically significant.

## 3. Results

### 3.1. Maximal Oxygen Uptake Confirmation

The participants obtained a Wmax equal to 315.9 ± 38.9 (W) (95% CI 294.6–337.1 W) during the IET_RAMP_. The duration of the ramp test was 18:57 ± 2:03 (min:s) (95% CI 17:41–20:14 min:s, and for the VER_85_, was 7:31 ± 1:30 (min:s) (95% CI 6:43–8:19 min:s) (*p* < 0.001, t = 19.66, ES = 5.73). The work performed during the IET_RAMP_ was 182.3 ± 46.8 (kJ) (95% CI 157.4–207.2 kJ). In the VER_85_, it was 60.1 ± 13.9 (kJ) (95% CI 52.6–67.5 kJ) (*p* < 0.001, t = 11.16, ES = 3.54), which was equal to 34.4 ± 10.2% of the total work performed during the IET_RAMP_.

**Figure 2:**
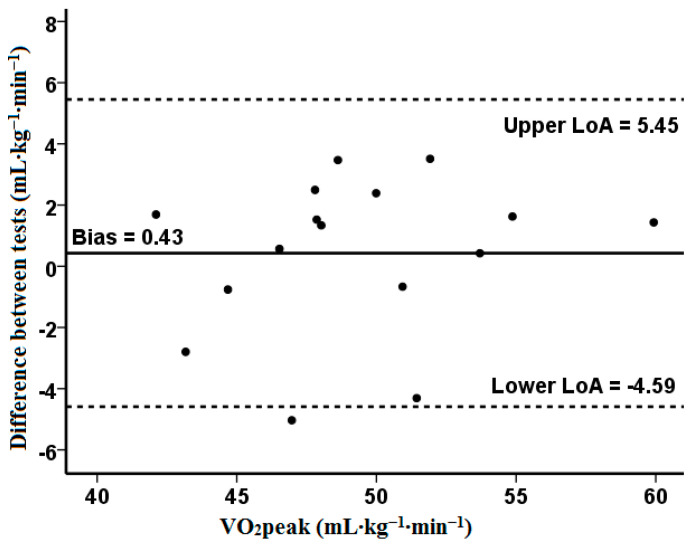
The Bland–Altman plot was used to define standard deviation, precision, and limits of agreement between the measurements of VO_2_peak from the two tests. The measure differences (*y*-axis) are delineated as a two-measure mean function (*x*-axis) at VO_2_peak. The horizontal solid line represents the mean difference between two measures (i.e., deviation). The two horizontal dotted lines represent the upper and the lower limit of agreement (1.96·SD) of the mean difference between VO_2_peak in IET_RAMP_ and VER_85_.

**Figure 3:**
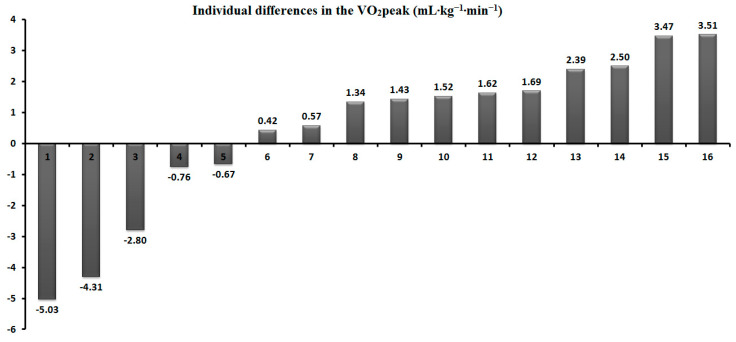
Individual differences in the VO_2_peak between IET_RAMP_ and VER_85_.

Table 2 shows that the peak oxygen uptake did not show significant differences between the tests (*p* = 0.51). During the VER_85_, the VCO_2_ was significantly lower (*p* < 0.05, t = 2.20, ES = 0.34), similar to HRmax (*p* < 0.05, t = 2.36, ES = 0.23). The CV was 3.1%, and the Bland–Altman analysis revealed a high agreement and a small bias for the VO_2_peak (0.43). One outlier was identified outside the lower limits of agreement (−4.59) (Figure 2). Moreover, five participants (31%) achieved a higher value during the VER_85_ (Figure 3). The peak oxygen uptake for the group that obtained it in the IET_RAMP_ (n = 11) was 51.1 ± 4.8 mL kg^–1^ min^–1^ (95% CI 47.8–54.3), and in the verification test (n = 5), it was 48.8 ± 3.9 mL kg^–1^ min^–1^ (95% CI 43.9–53.7) (*p* = 0.50).

### 3.2. Anaerobic Performance

The PPO measured in the WAnT was 865.0 ± 94.6 (W) (95% CI 814.6–915.3 W) and the rPPO was equal to 11.2 ± 0.7 (W·kg^−1^) (95% CI 10.8–11.6 W·kg^−1^). The total work performed was 19.4 ± 1.9 (kJ) (95% CI 10.8–11.6 kJ), and the fatigue index was equal to 27.8 ± 5.4 (%) (95% CI 24.9–30.6%). The total work performed during the IET_RAMP_ was correlated with TW_WAnT_ (r = 0.67, *p* < 0.01) and the PPO (r = 0.52, *p* < 0.05). There were no correlations between the level of anaerobic performance in the WAnT and the total work performed in the verification test.

Table 3 shows a comparison of the anaerobic performances between two groups depending on where the individuals obtained a higher peak oxygen uptake (VO_2_peak in IET_RAMP_ (n = 11) vs. VO_2_peak in VER_85_ (n = 5)). This division was used to test the second hypothesis and compare the anaerobic performances between these both groups. There were no significant differences in the PPO (*p* = 0.50), rPPO (*p* = 0.74), TW_WAnT_ (*p* = 0.36), or FI (*p* = 0.74) between these groups (Table 3).

### 3.3. Respiratory Muscle Strength

**Figure 4:**
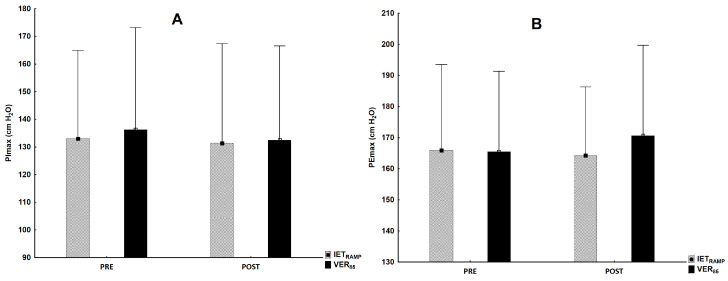
The level of inspiratory (**A**) and expiratory (**B**) muscle strength at rest and after completion of IET_RAMP_ and VER_85_.

There were no post-exercise changes in the inspiratory (F_1,30_ = 1.13, *p* = 0.30) and expiratory muscle strength (F_1,30_ = 0.38, *p* = 0.54) in relation to rest in the IET_RAMP_ and VER_85_ and between the tests for PImax_POST_ (F_1,30_ = 0.18, *p* = 0.68) and PEmax_POST_ (F_1,30_ = 41.0, *p* = 0.23) (Figure 4).

## 4. Discussion

The findings of this study indicate that the verification test performed at submaximal intensity (85% Wmax) to volitional exhaustion, on a different day to the IET_RAMP_, confirms the VO_2_max expressed as group mean. Another finding revealed here was the acceptable individual variation in VO_2_peak, with five subjects achieving a higher VO_2_peak during the VER_85_. Moreover, for the second hypothesis, we reported equivocal results. The positive correlations between the peak power output, the work performed in the WAnT, and the work performed in IET_RAMP_ indicate the importance of anaerobic performance. There were no differences in the anaerobic performance indicators between groups taking into account the test where individuals obtained a higher peak oxygen uptake, but a trend to lower mechanical indices occurred for the VER_85_ group. Thus, a verification test may be suggested for participants with a lower anaerobic mechanical performance. A lack of changes in the strength of the inspiratory and expiratory muscles indicates their marginal effect on VO_2_max measurements during the verification phase. This study is the first to provide the role of anaerobic mechanical performance and its influence on VO_2_max measurements during ramp incremental tests and verification tests with a simultaneous lack of post-test respiratory muscle fatigue.

Typically, in similar studies [16,17,18,19,20,21,22], the VO_2_peak between the incremental exercise test and verification test was compared as a group mean. Noakes [43] criticized this approach, stating that the verification of the VO_2_peak against that measured in an IET should be considered as an individual response. The use of the arithmetic mean for all participants in statistical analyses may not identify the individual variability in the VO_2_peak measurement between these tests. Our study confirms this statement, as the mean difference in the VO_2_peak between the IET_RAMP_ and VER_85_ was only 0.8% and was not significant. The coefficient of variation for VO_2_peak was 3.1% and was close to the most commonly used CV measures, which assume differences of 2–3% [29,44]. In other studies, some authors have utilized less restrictive criteria of ≤5–5.5% for the acceptable differentiation of measurements of physiological response [45,46]. Moreover, Villanueva et al. [19] reported a more significant agreement between the peak VO_2_ obtained in the incremental exercise test and the submaximal verification test (85% IET peak power) compared to the supramaximal test (110% IET peak power), which was analyzed by examining the limits of agreement and bias presented in Bland–Altman plots. Similarly, this is confirmed by the Bland–Altman analysis carried out in our study, which indicates the existence of a high agreement between the verification phase and the ramp test for VO_2_max, evaluating with one outlier. Although the need for the verification test seems questionable according to some researchers, additional tests prove helpful in assessing the individual variability in VO_2_peak [21]. Murias et al. [16] concluded that performing an additional verification test may allow for measuring a higher VO_2_peak value, which, for some reason, cannot be achieved during the IET. This is consistent with our results, because the highest VO_2_peak (including both tests) was 1% higher than that measured only during the IET_RAMP_ (50.3 vs. 49.5 mL·kg^–1^·min^–1^). Thus, this justifies conducting a test to verify the VO_2_max measured in an IET, as previously postulated by Poole and Jones [10].

The results from this study should also be evaluated considering the context of the day when the verification test was performed (after IET or on another day). Previous reports have shown that these tests at submaximal intensity (80–90% Wmax) were performed on the same day in groups of young and older people [16,19,45,46,47] or on a different day [20,48]. The results from our study are consistent with those published by Murias et al. [16] and Villanueva et al. [19], who also applied the submaximal load verification test with 85% of the maximal power obtained in the IET. Villanueva et al. [19] compared two verification phases with intensities of 85% and 110% Wmax from the ramp incremental test (with a linear increasing load) was performed 10 min after the IET. These authors suggested that submaximal exercise at a constant intensity (85% Wpeak) verifies VO_2_max more frequently in elderly participants [19]. However, in the studies mentioned above, verification tests were carried out several minutes after the completion of the incremental exercise test. The results from our study are consistent with those reported by Sawyer et al. [20] and Hebisz et al. [18], who also used verification tests on another day. This approach was suggested due to a lower level of fatigue, as the possibility of a longer effort and obtaining the actual VO_2_peak on a separate day may be preferable for participants with a lower physical capacity or those with overweight or obesity [18,49,50]. This justifies further research on the methodological aspects for the application of a verification phase and a detailed explanation of the impact of potential factors, e.g., the longer duration of the VER test [22] performed on a different day or the potential role of anaerobic performance.

Hill et al. [51] suggested that at least a 2 min time to exhaustion may be required to induce VO_2_max. Exercise at an intensity above the critical power should lead to the achievement of the VO_2_peak [16,52]. This justifies using a verification phase with submaximal intensity and an open-loop task [22,23]. The mean time of the verification phase measured by us was longer than that in the previously mentioned studies and amounted to 7:31 ± 1:30 (min:s) [15,16,19,20]. This may be related to the level of physical fitness and the implemented procedures, e.g., performing the VER on a different day. Additionally, the duration of our both tests was consistent with the suggestion of Midgley et al. [53] on measuring VO_2_max in cycle ergometer tests lasting from 7 to 26 min. The criteria for establishing the maximum effort and VO_2_max were executed in both tests, although the peak HR during the VER was significantly lower than that during the ramp IET. But it is noteworthy that, even if statistically different, the 2 (beats·min^–1^) found in our study might be seen as a clinically meaningful difference. Similar results were obtained when comparing two incremental ramp protocols, where a considerably lower HRmax was obtained in the shorter test [54]. According to Boudet et al. [55], more extended and high-intensity exercise generates an additional increase in catecholamines and sympathetic response. According to Astorino and White [29], the lack of differences in peak HR values indicates the usefulness of this criterion when verifying the peak oxygen uptake. On the other hand, it was considered that, if the difference between HRmax during the incremental and verification phases is ≤4 beats per minute, this is regarded as sufficient evidence for maximal effort [56]. Further research should consider other central and peripheral factors that determine the VO_2_max and their impact on the individual variability in VO_2_peak.

The incremental exercise test and some types of verification tests are open-loop tasks performed until exhaustion. Therefore, we assumed that the level of the anaerobic performance would determine the ability to tolerate the exercise intensity and achieve VO_2_max [24,25]. Our findings revealed similar post-exercise lactate concentrations and blood pHs measured in both tests, indicating the achievement of a limited anaerobic performance and level of acidosis, which is a factor causing skeletal muscle fatigue, leading to limitations in continuing exercise and voluntary exhaustion [57]. We also examined whether the participants who obtained VO_2_peak in the VER_85_ (n = 5) differed in their anaerobic performance and VO_2_peak compared to subjects who “preferred” the IET_RAMP_ (n = 11). Interestingly, all measured parameters in the WAnT were similar, without any significant differences. Moreover, in this study, we used a novel approach and compared the parameters depending on the test type. In other studies, different approaches were carried out, e.g., Astorino and White [29] compared the anaerobic performances of participants who confirmed or did not confirm VO_2_max in the verification test. It was shown that subjects with a lower level of cardiovascular fitness, without experience in reaching maximal effort and not adapted to exceed the upper limits of their exercise tolerance, might interrupt the IET before reaching VO_2_max due to symptoms related to fatigue [10,58]. Alternatively, the central governor model of exercise regulation [59] explains that brain activity may weaken exercise tolerance by reducing motor unit recruitment during demanding exercise. Moreover, we found significant correlations between the work performed in the IET_RAMP_ and the peak power output and total work performed in the WAnT. Similar relationships did not occur in the case of VER_85_, which indicates that anaerobic performance (expressed as power and the work measured in the WAnT), to a greater extent, determines the possibility of continuing effort until exhaustion, overcoming mechanical resistance and performing greater work in the ramp incremental test. It seems that subjects with a lower anaerobic performance (mechanical power) may prefer the submaximal verification test with a constant load than the increasing workload during the ramp incremental test. However, further research should comprehensively explain the role of anaerobic performance.

The third tested hypothesis was the examination of the occurrence of post-exercise respiratory muscle fatigue after the tests. Previously, it was well-documented that respiratory muscle fatigue occurs during heavy exercise (>80% VO_2_max) lasting 8–10 min [60,61], which may limit the possibility to continue strenuous exercise (like in the IET and VER tests). Oueslati et al. [31] reported post-exercise fatigue as decreased respiratory muscle strength after an incremental exercise test on a treadmill and cycle ergometer in runners and cyclists. The results of our study are contrary to these findings, because the respiratory muscle strength did not differ significantly after the IET_RAMP_ and VER_85_. One of the possible explanations for these discrepancies is the baseline level, which was similar to runners but lower for cyclists than the level reported in our study. It is possible that the exercise modality, a standing posture, could induce a different recruitment pattern of the respiratory muscles between running and cycling [31]. For example, Tanner et al. [62] showed differences between the end-inspiratory (6.24 ± 0.88 vs. 5.90 ± 0.74 L) and end-expiratory (3.40 ± 0.53 vs. 3.21 ± 0.55 L) lung volumes for the same group of participants in a maximal cycling test compared to a running test. In summary, the maximal lung ventilation was very similar in both tests and there were no fatigue symptoms, so we conclude that these factors do not influence VO_2_peak during verification tests.

The limitation of our study is that its results are relevant to young, active men, not professional athletes or other groups (women, older people, and those with obesity). Therefore, further studies should include women as well as elderly people due to the importance of tests for assessing cardio-respiratory efficiency. The study’s limitations also include the fact that this test is specific for incremental exercise test on a cycle ergometer. We only assessed anaerobic mechanical performance, but metabolic indices should also be evaluated, such as maximal accumulated oxygen deficit. On the other hand, we implemented a resistance of 7.5% of body weight during the WAnT. However, mechanical anaerobic power could be evaluated by a more specific force–velocity test using the optimal load [63]. It is noteworthy that other methods could be implemented for respiratory muscle strength examination. Further analysis should include the above-mentioned suggestions.

## 5. Conclusions

These findings indicate that a verification test of submaximal intensity (85% Wmax) performed on a different day confirms VO_2_max and emphasized the individual inter-subject variability in the male subjects. It should be taken into account that nearly one-third of the participants had a higher peak oxygen uptake measured in the verification test. Scientists should further investigate which test protocols could minimize the differences between the VO_2_peak attained in IET and VER tests. Participants with higher levels of anaerobic performance may perform greater work and are likely to achieve a higher VO_2_peak during the ramp IET. Whether the role of anaerobic performance can explain individual variability should be comprehensively examined in the future investigations. We also concluded that the applied tests do not cause fatigue of the respiratory muscles and, therefore, have no effect on the diagnosed VO_2_peak. Thus, the proposed verification test can be an alternative that is less physically demanding in order to achieve the peak physiological parameters.

## Figures and Tables

**Table 1:** Participants’ characteristics (n = 16) ($\bar{x}\;\pm \;SD$).

Variables	$\bar{x}\pm SD$
Age (years)	22.7 ± 2.4
Body height (cm)	178.0 ± 7.4
Body mass (kg)	77.4 ± 7.3
%FAT	16.4 ± 3.6
PA (h per week)	7 ± 1
SP (mm Hg)	125 ± 9
DP (mm Hg)	71 ± 7
FVC (L)	7.5 ± 1.5
FEV_1_·FVC^–1^ (%)	73.9 ± 8.2
Hb (g·dL^−1^)	14.8 ± 1.1
Ht (%)	43.1 ± 7.8

%FAT—the percentage of fat in total body weight, FVC—forced vital capacity in first second, FEV_1_·FVC^–1^—Tiffeneau index, PA—physical activity, SP—systolic pressure, DP—diastolic pressure, Hb—hemoglobin concentration, and Ht—hematocrit.

**Table 2:** Comparison of the mean ($\bar{x}$ ± *SD*) and 95% CI physiological, biochemical, and psychological responses between both tests.

Variables	IET_RAMP_		VER_85_	
	$\bar{x}$ ± *SD*	95%CI	$\bar{x}$ ± *SD*	95%CI
Rfmax (breath·min^–1^)	55.2 ± 16.1	46.7–63.8	53.9 ± 11.3	47.9–59.9
VTmax (L)	3.2 ± 0.6	2.9–3.5	3.2 ± 0.5	2.9–3.5
VEmax (L·min^–1^)	148.9 ± 26.7	134.7–163.1	148.1 ± 21.6	136.5–159.8
VCO_2_max (L·min^–1^)	4.3 ± 0.6	4.0–4.6	4.1 ± 0.4 *	3.9–4.4
$\dot{V}$O_2_peak (mL·kg^–1^·min^–1^)	49.5 ± 5.0	46.9–52.1	49.1 ± 4.5	46.7–51.4
RERmax	1.14 ± 0.03	1.12–1.16	1.15 ± 0.05	1.12–1.16
HRmax (beats·min^–1^)	193 ± 9	188–198	191 ± 9 *	186–196
SV_1_ (mL)	125.4 ± 19.3	115.1–135.6	119.9 ± 21.1	108.7–131.1
SV_2_ (mL)	122.1 ± 15.2	114.0–130.2	122.5 ± 13.8	115.2–129.8
VO_2_·HR-1 (mL·beats^–1^)	20.0 ± 2.5	18.7–21.3	20.6 ± 3.5	18.7–22.4
[La^−^] (mmoL·L^–1^)	12.1 ± 1.5	11.3–12.9	12.4 ± 1.6	11.6–13.3
pH post	7.21 ± 0.05	7.19–7.24	7.22 ± 0.04	7.19–7.24
RPE (6–20)	19 ± 1	18–19	18 ± 1	18–19

Values presented as means ± SD with 95% confidence intervals. Rfmax–maximal respiratory frequency, VTmax—maximal tidal volume, VEmax—maximal minute ventilation, VCO_2_max—maximal carbon dioxide production, $\dot{V}$O_2_peak—peak oxygen uptake, RERmax—maximal respiratory exchange ratio, HRmax—maximal heart rate, SV_1_—stroke volume estimated based on blood pressure, SV_2_—stroke volume from Fick’s equation, $\dot{V}$O_2_·HR^−1^—oxygen pulse, [La^−^]_POST—_post-exercise lactate concentration, RPE—rate of perceived exertion, and *—significant difference *p* ≤ 0.05.

**Table 3:** Comparison of the anaerobic performance of the group that obtained peak oxygen uptake in the IET_RAMP_ and the VER_85_ ($\bar{x}$ ± *SD*) and 95% CI.

Variables	$\dot{V}$O_2_peak in IET_RAMP_ (n = 11)		$\dot{V}$O_2_peak in VER_85_ (n = 5)	
	$\bar{x}$ ± *SD*	95%CI	$\bar{x}$	95%CI
PPO (W)	878.9 ± 110.4	804.7–953.1	834.3 ± 36.4	789.1–879.4
rPPO (W·kg^−1^)	11.3 ± 0.8	10.7–11.8	11.0 ± 0.5	10.4–11.7
TW_WAnT_ (kJ)	19.7 ± 2.1	18.3–21.2	18.6 ± 0.9	17.5–19.7
FI (%)	27.4 ± 5.6	23.6–31.3	28.6 ± 5.5	21.8–35.5

PPO—peak power output, rPPO—peak power output per kilogram, TW_WAnT_—total work in the Wingate Anaerobic Test, and FI—fatigue index.

## Data Availability

Data are available upon request to author KM.
